# IVF outcome with a high level of AMH: a focus on PCOS versus non-PCOS

**DOI:** 10.1186/s12905-022-01756-4

**Published:** 2022-05-14

**Authors:** R. Muharam, Yohanes Danang Prasetyo, Kevin Ardito Prabowo, Yuannita Ika Putri, Mila Maidarti, Andon Hestiantoro

**Affiliations:** 1 Division of Reproductive Immunoendocrinology, Department of Obstetrics & Gynecology, Faculty of Medicine, University of Indonesia–Cipto Mangunkusumo National Hospital, Jl. Pangeran Diponegoro No.71, Kenari, Kec. Senen, Kota Jakarta Pusat, Daerah Khusus Ibukota Jakarta, 10430 Indonesia; 2 Department of Obstetrics and Gynecology, Faculty of Medicine, University of Indonesia–Cipto Mangunkusumo National Hospital, Jl. Pangeran Diponegoro No.71, Kenari, Kec. Senen, Kota Jakarta Pusat, Daerah Khusus Ibukota Jakarta, 10430 Indonesia

## Abstract

**Background:**

The purpose of this research was to investigate whether high AMH levels in PCOS patients resulted in different IVF outcomes compared to those in non-PCOS patients.

**Methods:**

A retrospective cohort study was conducted involving 238 women undergoing IVF who had AMH levels > 4 ng/ml. Participants were divided into two groups: PCOS and non-PCOS.

**Results:**

The median AMH level was significantly higher in the PCOS group (7.59 ± 4.61 ng/ml vs. 5.91 ± 2.22 ng/ml, p < 0.001). The PCOS group required less gonadotropin but yielded more oocytes after stimulation. Significantly more participants from the PCOS group (41.5% [n = 39]) developed a hyperresponse to ovarian stimulation compared to the non-PCOS group (26.4% [n = 38]) (OR = 1.978, 95% CI 1.138–3.488; p = 0.015).

**Conclusion:**

There were significant differences in terms of total doses of gonadotropin and the number of oocytes retrieved in the PCOS and non-PCOS groups. Women with PCOS and high AMH levels have a higher risk of hyperresponse after ovarian stimulation than women without PCOS.

## Introduction

The use of anti-Mullerian hormone (AMH) analyses to predict ovarian response to stimulation has been widespread. A high level of AMH (above 4–5 ng/ml) has been associated with a higher risk of hyperresponse and ovarian hyperstimulation syndrome (OHSS) occurrence [1]. Hyperresponse is characterized by the number of oocytes retrieved ≥ 20, development of OHSS, cycle cancellation due to hyperresponse, or combination of these conditions [2].

AMH is secreted from preantral and small antral follicles. It has also been associated with hyperandrogenism [3]. Due to these characteristics, polycystic ovary syndrome (PCOS) patients tend to have higher AMH levels. Wiweko et al. reported that patients with a mean AMH level ≥ 4.45 ng/ml have a 9.35 times higher likelihood of developing PCOS [4]. However, high levels of AMH have also been found in non-PCOS patients. In in vitro fertilization (IVF), AMH levels are correlated with ovarian reserve and response to controlled ovarian stimulation. AMH also appears to be correlated with IVF outcomes, such as implantation, clinical pregnancies, and live birth rates, despite conflicting results [3]. AMH levels can also predict ovarian response to exogenous follicle-stimulating hormone (FSH). Many studies have been conducted to identify the correct FSH doses during controlled ovarian stimulation (COS) in IVF, with markers of ovarian reserve as part of the considerations. However, the studies were not adequate for women with PCOS, particularly those with higher AMH levels [5–7]. Studies that attempt to observe the difference in the high AMH state in PCOS and non-PCOS patients and its effect on IVF outcomes are still very limited.

Therefore, this study aimed to determine whether high AMH levels in PCOS and non-PCOS patients might result in differences in their IVF outcomes.

## Methods

### Study population

We conducted a retrospective cohort study of eligible women who underwent an IVF cycle in our fertility clinic between January 2013 and June 2020. The data were obtained from medical records. Approval for this study was acquired from the Health Research Ethics Committee—University of Indonesia and Cipto Mangunkusumo Hospital. The inclusion criteria used were as follows: (1) serum AMH level > 4 ng/ml and (2) underwent the GnRH antagonist protocol. The exclusion criteria used were as follows: (1) frozen embryo transfer (FET) cycle, (2) cycles canceled prior to oocyte pick up, and (3) incomplete data.

The study population was divided into two groups: the PCOS group and the non-PCOS group. The diagnosis of PCOS was established based on the Rotterdam criteria when at least two of the following three criteria existed: oligomenorrhea or amenorrhea, clinical hyperandrogenism and/or hyperandrogenemia, and polycystic ovaries.

### Clinical and laboratory protocols

Serum AMH levels were measured prior to initiating the COS protocol in each patient. All AMH measurements were performed at our clinic’s laboratory.

Initial gonadotropin dosages were based on patient age, weight, antral follicle count, and previous response to stimulation, if any. COS was achieved with gonadotropins (Gonal F; EMD-Serono, Switzerland). Ovulation was suppressed using 0.25 mg Cetrotide (Cetrorelix; EMD-Serono, Switzerland).

rhCG (Ovidrel; EMD-Serono, Switzerland) was used as the ovulation trigger. The rhCG trigger was generally given when the two lead follicles attained a mean diameter > 18 mm. Oocyte retrieval was performed under conscious sedation and transvaginal ultrasound (TVUS) guidance with a 30-cm 16 G oocyte aspiration needle 35–36 h after the ovulatory trigger. Luteal support with 50 mg of intravaginal progesterone (Crinone; Merck Serono, Denmark) was initiated daily the day after retrieval.

Fertilization of oocytes was performed with conventional insemination or intracytoplasmic sperm injection (ICSI) based on the couple’s history and the male partner’s semen analysis. All embryos were cultured using in-house culture media. Cleavage embryos were assessed on Day 2 (44–46 h after insemination or sperm injection) and Day 3 (66–72 h after insemination or sperm injection). Embryos were graded based on the criteria described by Veeck [19]. Embryo transfers were performed with Wallace catheters (Smiths Medical, OH, USA) at approximately 1 cm less than the uterine depth identified at prior trial transfer.

### Outcome variables

Demographic characteristics included AMH level, age, body mass index (BMI), type of infertility, duration of infertility, and infertility diagnosis. The primary IVF outcomes measured were biochemical pregnancy (BP) rate, clinical pregnancy (CP) rate, ongoing pregnancy (OP) rate, and OHSS occurrence.

Secondary outcomes measured COS parameters and outcomes, including total days of COS, total gonadotropin administered, endometrial thickness, total number of oocytes retrieved, mature oocytes rate, and fertilization rate. The fertilization rate was defined as the percentage of transformation of inseminated oocytes into two pronuclei (2PN) embryos.

Embryo evaluation was performed according to the Graduated Embryo Score (GES) criteria (Fisch et al., 2001). Patients who had at least three embryos with scores equal to or greater than 80 points according to the criteria on Day 3 were considered eligible to undergo five days of embryo culture. On Day 3 of evaluation, embryos that met the following criteria were considered good-quality embryos: (1) less than 25% fragmentation, (2) stage-specific cell size for the majority of cells, and (3) no multinucleation. The Gardner blastocyst scoring system was used to evaluate the quality of blastocysts on Day 5 of embryo culture.

### Statistical analysis

All statistical analyses were performed using SPSS version 25 (IBM Corp; Armonk, NY, USA). Categorical variables are presented as the number of cases and corresponding percentages. Continuous variables are presented as the mean ± SD. Mean differences between the two groups were compared using Student’s t test or the Mann–Whitney test, as appropriate. A p < 0.05 was considered statistically significant.

## Results

A total of 238 women aged 24–41 years met the criteria and were enrolled in the study. The mean age of the non-PCOS group was found to be higher (34.10 ± 4.45 vs. 32.73 ± 3.63, p = 0.01). The median AMH level was significantly higher in the PCOS group (7.59 vs. 5.91, p < 0.001). No other characteristics showed a significant difference between the two groups (Table 1).

**Table 1:** Patient characteristics

Variables	PCOS (n = 94)	Non-PCOS (n = 144)	p value
Age (years)	32.73 ± 3.63	34.10 ± 4.45	0.010^b^
BMI (kg/m^2^)	23.21 (5.02)	23.62 (5.34)	0.784^a^
Type of infertility (n [%])			
Primary Secondary	80 (85.1%) 14 (14.9%)	122 (84.7%) 22 (15.3%)	
Duration of infertility (years)	5 (4)	6 (6)	0.161^a^
Infertility diagnosis (n [%])			
Male factor Tubal Ovulatory Endometriosis Other	–	75 (52.1%) 15 (10.4%) 1 (0.7%) 12 (8.3%) 41 (28.5%)	
AMH (ng/ml)	7.59 (4.61)	5.91 (2.22)	< 0.001^a^

^a^Mann–Whitney test

^b^Independent t test

Normally distributed continuous data are expressed as the mean ± standard deviation, whereas nonnormally distributed data are expressed as the median (interquartile range); categorical data are expressed as n (%)

From the COS process, we found that the PCOS group required less gonadotropin (2085 vs. 2550, p = 0.001). However, despite a lower dosage of gonadotropin administered, more oocytes were retrieved in the PCOS group after stimulation (17 vs. 14, p = 0.002) (Table 2).

**Table 2:** COS parameters and outcomes

Variables	PCOS (n = 94)	Non-PCOS (n = 144)	p value
Total days of COS (days)	10 (2)	11 (2)	0.177^a^
Total gonadotropin doses (IU)	2085 (938)	2550 (1706)	0.001^a^
Endometrial thickness (mm)	11.7 (2.43)	12 (3.78)	0.131^a^
Number of oocytes retrieved (n)	17 (12)	14 (10)	0.002^a^
Mature oocytes rate (%)	83.30 (18.8)	83.65 (18.3)	0.142^a^
Fertilization rate (%)	75.0 (23.3)	71.4 (25.7)	0.995^a^
OHSS (n [%])	5 (5.3%)	5 (3.5%)	0.448*
Response (n [%])			0.077*
• Poor response • Normal response • Hyperresponse	0 (0%) 55 (58.5%) 39 (41.5%)	2 (1.4%) 104 (72.2%) 38 (26.4%)	

^a^Mann–Whitney test

^*^Chi-square test

Nonnormally distributed continuous data are expressed as the median (interquartile range); categorical data are expressed as n (%)

In the PCOS group, two patients had mild PCOS, and three had severe OHSS. Meanwhile, in the non-PCOS group, four patients had mild OHSS, and one had severe OHSS. Patients with severe OHSS had to have their cycle canceled, and the embryo had to be frozen. Two other patients in the PCOS group also chose to have their embryos frozen due to nonclinical indications. Hyperresponse cases were found to be significantly higher in the PCOS group (41.5% vs. 26.4%, p = 0.015), with the OR = 1.978 (95% CI 1.138–3.488) (Table 2).

We found no statistically significant difference between the two groups in terms of embryo quality parameters (Table 3). Pregnancy rates also showed no significant difference between the two groups (Table 3).

**Table 3:** Embryo and IVF outcomes

Variables	PCOS (n = 89)	non-PCOS (n = 143)	p value
Number of transferred embryo (n)	2 (1)	2 (1)	0.433^a^
Good-quality embryo (n [%])	82 (87.2%)	137 (95.1%)	0.237*
Day 5 transfer (n [%])	43 (45.7%)	59 (41.0%)	0.292*
Cumulative BP rate (%)	58 (61.7%)	80 (55.6%)	0.164*
Cumulative CP rate (%)	43 (45.7%)	55 (38.2%)	0.140*
Cumulative OP rate (%)	25 (26.6%)	49 (34.0%)	0.326*

^a^Mann–Whitney test

*Chi-square test

Nonnormally distributed continuous data are expressed as the median (interquartile range); categorical data are expressed as n (%)

Additionally, we also tried to determine the cutoff value of AMH to predict the hyperresponse in both groups. The receiver operating charateristic (ROC) curve was plotted for each group, and the area under the curve (AUC) was recorded. In the PCOS group, the AUC value was 0.626 (95% CI; sensitivity: 71.8%; specificity: 52.7%), and in the non-PCOS group, the AUC value was 0.617 (95% CI; sensitivity: 71.1%; specificity: 46.2%). AUC values of 0.626 and 0.617 (in the PCOS and non-PCOS groups, respectively) indicated poor predictive quality.

## Discussion

Our results show that there were significant differences in terms of total doses of gonadotropin and the number of oocytes retrieved in the PCOS and non-PCOS groups. The PCOS group required less gonadotropin but yielded more oocytes after stimulation. However, in terms of pregnancy outcomes and embryo quality, there were no significant differences between the two groups.

Among the patient characteristics, we found a significant difference in the median age and AMH level between the two groups. These findings are similar to those of previous studies. For example, Lauritsen et al. (2014) reported a lower PCOS prevalence with increasing age [8]. This might be caused by the decrease in ovarian reserve or antral follicles as a woman ages [9].

Higher AMH levels in PCOS patients have also been reported in numerous studies [4, 10–13]. This might be caused by the increased synthesis and secretion of AMH by preantral and small antral follicles [3]. The level of AMH increases with the antral follicle count (AFC) at a consistent rate of 0.2 ng/ml per follicle [14]. In addition, granulosa cells in the follicles of PCOS patients have been shown to produce 75-fold more AMH than normal cells [15].

From the IVF process, we found that the PCOS group received a significantly lower gonadotropin dose during COS. However, the number of oocytes retrieved was found to be higher. Moreover, the number of hyperresponders in the PCOS group was also significantly higher than that in the control group. Although all the study subjects received the antagonist protocol, different doses might have been given. Following our clinical protocol, the protocol and doses were determined based on patient age, BMI, AFC, and previous response to stimulation, if any.

A study conducted by Di Paola et al. identified the correct FSH doses during COS in IVF, with markers of ovarian reserve as part of the considerations. However, the protocol was not adequate for women with PCOS, particularly those with higher AMH levels [5]. Indeed, the major problem of gonadotropin stimulation in PCOS patients is the increased risk of multiple pregnancy and OHSS. Therefore, low-gonadotropin stimulation has been introduced and recently has been accepted as the best practice for PCOS patients, especially for those who have clomiphene citrate (CC) resistance [16, 17]. In the last few years, a more sophisticated method has been introduced. Individualized COS (iCOS) involves identifying high-risk patients through various biomarkers, with AMH and AFC seeming to be promising variables [2, 18]. Few studies have reported that the use of iCOS led to significantly lower OHSS occurrence [19, 20].

Our PCOS group showed significantly higher average numbers of retrieved oocytes, despite lower administered gonadotropin doses. Various studies have effectively documented similar findings. Sahu et al. reported that despite a significantly lower total gonadotropin dose, PCOS and PCO-only groups yielded more oocytes compared to controls [21]. However, they also found no differences in oocyte maturation, embryo quality, implantation, or pregnancy rate. We discovered the same circumstances in our study. Swanton et al. also reported significantly more oocytes retrieved in PCOS and PCO-only groups (n = 14.2 and n = 16.2, respectively) compared to controls (n = 10.5). They also found significantly more severe OHSS cases in the PCOS and PCO-only groups, similar to our findings.

In this study, although the difference was nonsignificant, pregnancy outcomes were poorer in the PCOS group than in the non-PCOS group. A recent review by D’Alterio et al. concluded that OHSS and the greater proportion of multiple pregnancies in women with PCOS could explain the lower pregnancy outcomes reported. Insulin resistance, elevated BMI, and androgen concentration are correlated with adverse pregnancy outcomes in women with PCOS. Insulin resistance disturbs early placentation. Moreover, insulin resistance and hyperadrogenism cause chronic inflammation that could inhibit implantation. Increased miscarriage rates are also observed in women with PCOS with insulin resistance, hyperinsulinemia, and elevated body weight [22].

When we tried to determine a cutoff value of AMH levels for hyperresponse prediction, it resulted in a poor predictive value. This is presumably the result of the heterogenicity of each antral follicle. While AMH production is high, each follicle’s threshold to respond to ovarian stimulation might vary in women with PCOS. Kim et al. analyzed the correlation between AMH level multiples of median (AMH-MoM) and ovarian sensitivity to gonadotropin stimulation. They found that ovarian sensitivity was not correlated with the AMH-MoM value and even tended to decrease with an increasing AMH-MoM level [23]. These findings might suggest that the use of basal serum AMH levels as a predictor of ovarian response should be carefully applied, considering other factors in patients.

This study has some limitations. Due to its retrospective design and relatively small number of patients, our study had low power. Moreover, women with various PCOS phenotypes were recruited for the study, which might have played a role in PCOS patients’ response to gonadotropin stimulation. More comprehensive factors and data must be included to further investigate the underlying causes associated with OHSS in PCOS patients.

## Conclusion

There are significant differences in terms of total doses of gonadotropin and number of oocytes retrieved in the PCOS and non-PCOS groups. The PCOS group required less gonadotropin but yielded more oocytes after stimulation. Even though both groups had high AMH levels, women with PCOS still had a higher risk of hyper-response after ovarian stimulation. Further investigation of PCOS pathophysiology and other factors associated with it need to be done in the future.

## Data Availability

The data that support the findings of this study are available from the corresponding author on reasonable request.
